# Characteristics and Weight Loss Practices From a Cohort of 20,000 Patients Using Direct-to-Consumer Telehealth: Observational Cross-sectional Study

**DOI:** 10.2196/40062

**Published:** 2023-01-05

**Authors:** Deborah Bade Horn, Elizabeth Pash, Megan S Zhou, Lauren Broffman, Damian Bialonczyk, Tzvi Doron, Elaine Chiquette

**Affiliations:** 1 Center for Obesity Medicine and Metabolic Performance University of Texas Bellaire, TX United States; 2 Gelesis Boston, MA United States; 3 Ro New York, NY United States

**Keywords:** obesity, preobesity, overweight, weight management, direct-to-consumer telehealth, oral superabsorbent hydrogel

## Abstract

**Background:**

Despite the increasing prevalence of obesity, the use of pharmacotherapy treatment remains low. Telehealth platforms have the potential to facilitate access to pharmacotherapy interventions, but little is known about telehealth patients.

**Objective:**

This study describes a large patient population taking Plenity, an oral superabsorbent hydrogel (OSH) used in the treatment of excess weight or obesity (BMI 25-40 kg/m^2^). The analysis compared differences in weight loss practices and in-person access to obesity care among telehealth patients with preobesity and obesity.

**Methods:**

This was a cross-sectional assessment of a random sample of 20,000 telehealth patients who completed a structured, web-based visit and received at least one prescription of OSH. Patients were eligible to receive care via telehealth if they were adults, were not pregnant, and had a BMI ≥25 kg/m^2^. During the visit, patients provided baseline health information including comorbidities, diet, and exercise habits. Their zip code of residence was used to determine their proximity to an obesity medicine provider. Descriptive statistical analysis and tests of differences (chi-square and 2-tailed *t* tests) were used to compare patients with preobesity (BMI 25-29.9 kg/m^2^) and obesity (BMI 30-40 kg/m^2^).

**Results:**

Most (15,576/20,000, 77.88%) of the cohort were female, with a mean age of 44 (SD 11) years and a mean BMI of 32.4 (SD 4.1) kg/m^2^. Among the cohort, 32.13% (6426/20,000) had preobesity, and 40.18% (8036/20,000) of all patients had ≥1 weight-related comorbidity. Almost all (19,732/20,000, 98.66%) patients attempted 1 weight loss method before OSH and half (10,067/20,000, 50.34%) tried ≥4 different methods. Exercise and low-calorie diets were the most attempted weight loss methods, and 28.76% (5752/20,000) of patients reported a prior prescription of weight loss medication. Patients with obesity were more likely than patients with preobesity to have previously tried commercial weight loss plans (7294/13,574, 53.74% vs 2791/6426, 43.43%; *P*<.001), specialized diets (8493/13,574, 62.57% vs 3799/6426, 59.12%; *P*<.001), over-the-counter supplements (6807/13,574, 50.15% vs 2876/6426, 44.76%; *P*<.001), and prescription weight loss medications (4407/13,574, 32.47% vs 1345/6426, 20.93%; *P*<.001). Females were more likely to seek treatment for preobesity (5332/15,576, 34.23% vs 1094/4424, 24.73% male; *P*<.001) and reported fewer comorbidities (5992/15,576, 38.47% vs 2044/4424, 46.2% male; *P*<.001), despite >90% of both sexes reporting the belief that excess weight negatively affected their health (14,247/15,576, 91.47% female participants, 4116/4424, 93.04% male participants). Moreover, 29.25% (5850/20,000) of patients lived in the same zip code and 85.15% (17,030/20,000) lived in the same county as an obesity medicine provider.

**Conclusions:**

Data from this large patient cohort supports the potential for telehealth to provide prescriptive weight management treatment to a population seeking care. Patients with preobesity are an undertreated population who actively seek new weight management options. Female participants sought weight management treatment earlier in the disease continuum than males, despite reporting fewer comorbidities.

## Introduction

### Epidemiology of Obesity

Excess weight, defined as a BMI >25 kg/m^2^, is associated with increased morbidity and mortality and contributes to nearly 4 million deaths globally per year [1,2]. Obesity-related diseases and comorbidities are associated with a loss of between 0.2 and 11.7 years of life, depending on an individual’s sex, race, BMI category, and age [3]. In the United States, approximately 70% of adults have preobesity or obesity, and this number is expected to rise to 80% by 2030 [4,5]. Despite the increasing prevalence of obesity and difficulty in losing and maintaining lost weight in many patients, the use of guideline-supported treatments, including pharmacotherapy, intensive behavioral counseling, and bariatric surgery, remains low [6]. Only approximately 1% to 2% of eligible patients with obesity are managed with pharmacotherapy [7-9]. Comparatively, 86% of eligible patients with type 2 diabetes are prescribed pharmacotherapy [9].

### Barriers to Effective Treatment

Several barriers exist for effective weight management in patients with preobesity and patients with obesity [10]. Weight-related health stigma is one such barrier that disproportionately affects people with obesity [11,12]. People with obesity who experience weight stigma report more health care avoidance, increased perceived judgment from physicians, lower frequency of routine checkups, less frequent listening and respect from providers, and lower quality of health care [13]. Limited access to health care providers with training in obesity medicine and interdisciplinary treatment teams (eg, behavioral therapists, dietitians, and health coaches) poses yet another significant barrier to effective care [14,15]. Among nonspecialist providers, discussions about obesity are often not initiated with patients primarily because of lack of time and higher priority issues, despite more than half of health care providers considering obesity at least as serious as most other health conditions [10]. Geographic barriers, particularly in rural areas, further reduce access to effective obesity care [14]. Close to 40% of excess weight related deaths and 36% of disability-adjusted life years can be attributed to preobesity (BMI 25-30 kg/m^2^), also known as overweight [1]. Despite this, weight management options for patients with preobesity are generally limited to lifestyle modifications, as prescriptive pharmacotherapy is only available for patients with obesity or those with a BMI ≥27 kg/m^2^ with at least one obesity-related comorbidity.

### Pharmacological and Telehealth Solutions

Telemedicine, as a means of delivering weight loss treatment, has been proposed to help address some of the existing barriers to care in people with preobesity and obesity [6,16,17]. Today, multiple telehealth platforms provide patients seeking weight management with increased access to physicians, advanced practitioners, and health coaches. Specifically, direct-to-consumer (DTC) telehealth is an increasingly popular source of health care uniquely suited to facilitate access to treatment for stigmatized conditions such as obesity [6].

Plenity, a nonsystemic, oral superabsorbent hydrogel (OSH), is Food and Drug Administration cleared as a prescription treatment indicated to aid weight management in adults with excess weight or obesity, with a BMI of 25 to 40 kg/m², when used in conjunction with diet and exercise [18]. OSH is available by prescription from a nationwide DTC telehealth platform, Ro. Using the telehealth platform, patients interested in obtaining a prescription for OSH can request a web-based consultation with a provider and, if eligible, receive therapy by mail. Uniquely, OSH can be prescribed for patients with preobesity irrespective of comorbidity status, differing from most prescription options for weight management, which are generally reserved for use in people with a higher BMI (≥30 or ≥27 kg/m^2^) and obesity-related comorbidities.

### Objective of the Study

The purpose of this analysis was to describe a population of patients prescribed OSH via a telehealth platform and to explore the differences in weight loss practices between patients with preobesity and patients with obesity in a large real-world cohort. We also aimed to evaluate the extent to which users of the DTC telehealth platform had geographic access to an obesity medicine specialist.

## Methods

### Study Design

This is a retrospective cross-sectional study that examined the electronic health records of 20,000 patients who were prescribed at least one dose of OSH. These 20,000 patients were randomly selected from a sampling frame of patients being treated for weight management on a single DTC platform who were prescribed Plenity, had received at least one prescription, and whose records had complete baseline health information.

### Telehealth Platform Overview and Participant Eligibility

To formally become a patient on the DTC platform, potential self-referred patients initiate a structured, dynamic intake form in which they report their height, weight, health history, comorbidity status, demographics, and other relevant information. The information provided in the intake form comprised the baseline information recorded in the electronic health records. BMI was calculated from the patients’ self-reported height and weight. As a quality measure, patients were also required to upload recent photographs of themselves. Patients were considered eligible to receive weight management care via telehealth if they were aged ≥18 years, were not pregnant, did not have any allergies to OSH or its ingredients, did not report a history of eating disorders, and had a minimum BMI of 25 kg/m^2^. Patients who did not meet these requirements were automatically exited from the platform. Health information collected from eligible patients was reviewed by a health care provider (Doctors of Medicine, Doctors of Osteopathic Medicine, Nurse Practitioners, and other Advanced Practice Providers) licensed to practice medicine in the state where the patient resided, who then determined whether treatment was appropriate. Patients in the sample were located in almost every state, as well as Washington DC. If a provider deemed treatment appropriate, they connected with patients via video call, phone call, or asynchronously using a secure store-and-forward messaging system to discuss a care plan and answer any questions. Providers could also use these means of communication to clarify any baseline information provided by patients deemed necessary to make clinical decisions. If a decision was made to prescribe, providers sent prescriptions to a mail-order pharmacy and provided patients with educational materials encouraging healthy changes to diet and regular exercise. Patients were able to contact their providers using a secure messaging system throughout the course of their treatment to ask questions or schedule additional synchronous visits. To renew their initial prescription, the patients were required to complete renewal visits during regularly scheduled intervals.

Only patients who were prescribed Plenity, had received at least one prescription, and whose records had complete baseline health information were included in the study. Patients were grouped as patients with preobesity (6426/20,000, 32.13%, BMI 25-29.9 kg/m^2^,) or patients with obesity (13,574/20,000, 67.87%, BMI 30-40 kg/m^2^,) based on their self-reported height and weight at baseline. Patients were grouped in this manner to address the novelty of prescription weight loss treatment for the patients with preobesity group, which warrants further investigation. Patients were not further subdivided into classes of obesity because the clinical indication for OSH was the same for all patients, regardless of BMI. In addition, the upper BMI limit of the clinical indication of OSH would restrict the analyses to Classes I and II.

### Access to Care Outcomes

To determine rurality, the patients were geolocated to the zip code of residence provided for medication shipment. These zip codes were cross-referenced with 2019 rural-urban commuting area codes. Zip codes categorized as 4–9 within the rural-urban commuting area system were defined as rural. To determine whether patients lived in a healthcare provider shortage area (HPSA), publicly available data on the 2021 HPSA classification were retrieved from the Health Resources and Services Administration website. Patient zip codes were geolocated within counties using the zip code to county crosswalk from the Housing and Urban Development website; patient zip codes were converted to zip code tabulation areas. Zip code tabulation areas that spanned multiple counties were assigned to the county where most residences were located. A list of counties categorized specifically as primary care HPSA was cross-referenced with patient counties to determine whether patients resided in primary care HPSA. The American Board of Obesity Medicine (ABOM) provider practice locations were extracted by matching the list of provider names and cities available on the ABOM website with the National Provider Index Registry.

### Statistical Analysis

Chi-square and 2-tailed *t* tests were used as tests of significance when comparing BMI groups and sex. Bonferroni corrections were included where appropriate to account for the bias introduced by multiple tests. All data cleaning and analyses were performed using R (version 4.1.2, R Foundation for Statistical Computing).

### Ethics Approval

This study was approved by the Biomedical Research Alliance of New York Institutional Review Board (number 21-12-274-599).

## Results

### Demographics

**Figure 1:**
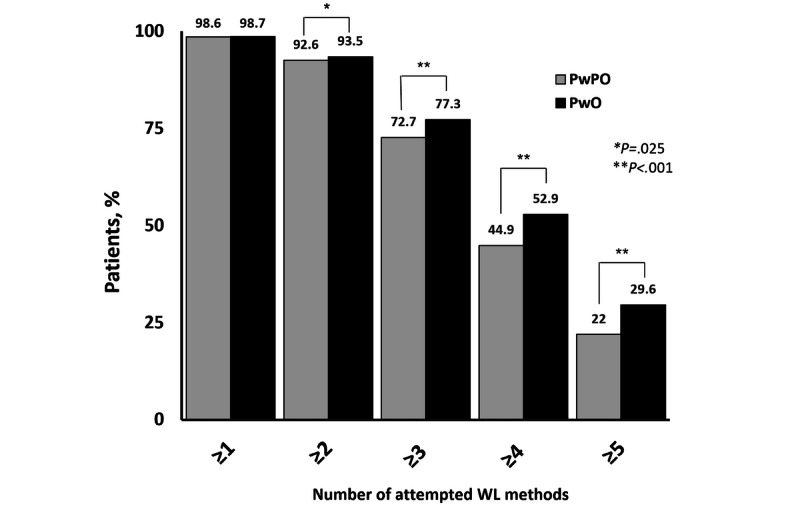
Number of weight loss (WL) attempts by BMI category. PwPO: people with preobesity; PwO: people with obesity.

Tables 1 and 2 present the demographic characteristics of the study population. Patients had a mean BMI of 32.4 kg/m^2^ and a median age of 43 (SD 11; range 19-86) years; approximately one-fourth (5407/20,000, 27.04%) of the sample population was >50 years, and 7.91% (1582/20,000) of the patients were ≥60 years old (Figure 1). The population was mostly female (15,576/20,000, 77.88%), and approximately one-third (13,574/20,000, 67.87%) of the cohort had preobesity. A question asking patients to report race or ethnicity data was added after initial baseline data were collected; subsequently, these data were only collected for 397 patients (of these patients, 6, 1.5% declined to answer and were excluded from the table). Of the patients who self-reported their race, approximately one-fourth (98/397, 24.7%) reported a race or ethnicity other than non-Hispanic White or White. When comparing geographic differences across patients, the data showed that 12.04% (2407/20,000) of all patients lived in a rural county.

We broke down the demographic characteristics across BMI (Table 1) and biological sex (Table 2) categories. Compared to patients with obesity, patients with preobesity were more likely to be female. No significant differences in race or ethnicity were detected between patients with preobesity and patients with obesity (*χ*^2^_5_=4.2; *P=*.47), but patients with obesity were slightly more likely to live in a rural area compared with patients with preobesity (*χ*^2^_1_=23.8; *P*<.001). No significant differences in age were observed between the 2 categories.

Significant differences were observed across biological sex, racial, and ethnic categories. Compared with males, females were more likely to self-report their race and ethnicity as non-Hispanic White (156/15,576, 78.4% female vs 143/4424, 72.2% male) and non-Hispanic Black (16/199, 8% female vs 7/198, 3.5% male), and were less likely to self-report as Hispanic or Latino (8/199, 4% female vs 26/198, 13.1% male). Females were also significantly more likely to live in rural areas (2020/15,576, 12.97% female vs 387/4424, 8.75% male; *χ*^2^_1_=57.6; *P*<.001).

**Table 1:** Sample demographics by BMI category.

Characteristic			Total patients (N=20,000)		Patients with obesity (n=13,574)		Patients with preobesity (n=6426)		Wilcoxon rank sum			Chi-square (*df*)			*P* value	
BMI, mean (SD)			32.4 (4.1)		34.6 (3.0)		27.8 (1.4)		87,226,524			N/A^a^			<*.*001	
Age, mean years (SD)			44 (11)		44 (11)		44 (11)		43,845,972			N/A			*.*54	
**Biological sex, n (%)**										N/A			142.3 (1)			*<*.001
	Female	15,576 (77.88)		10,244 (75.47)		5332 (82.98)										
	Male	4424 (22.12)		3330 (24.53)		1094 (17.02)										
**Race or ethnicity, n (%)**										N/A			4.2 (5)			*.*52
	Asian or Pacific Islander	19 (4.79)		12 (5.02)		7 (4.43)										
	Hispanic	34 (8.56)		24 (10.04)		10 (6.33)										
	Native American or American Indian	2 (0.50)		1 (0.42)		1 (0.63)										
	Non-Hispanic Black	23 (5.79)		17 (7.11)		6 (3.80)										
	Non-Hispanic White	299 (75.31)		174 (72.80)		125 (79.11)										
	Other	20 (5.04)		11 (4.60)		9 (5.70)										
**Geography, n (%)**										N/A			23.8 (1)			*<*.001
	Rural	2407 (12.04)		1739 (12.81)		668 (10.40)										

^a^N/A: not applicable.

**Table 2:** Sample demographics by biological sex.

Characteristic			Total patients (N=20,000)		Female (n=15,576)		Male (n=4424)		Wilcoxon rank sum		Chi-square *(df)*		*P* value
BMI, mean (SD)			32.4 (4.1)		32.3 (4.2)		32.9 (3.8)		31,109,884		N/A^a^		<.001
Age, mean years (SD)			44 (11)		44 (11)		44 (11)		34,733,596		N/A		.41
**Race or ethnicity, n (%)**									N/A		N/A		.02
	Asian or Pacific Islander	19 (4.79)		8 (4.02)		11 (5.56)							
	Hispanic	34 (8.56%)		8 (4.02)		26 (13.13)							
	Native American or American Indian	2 (0.50)		1 (0.50)		1 (0.51)							
	Non-Hispanic Black	23 (5.79)		16 (8.04)		7 (3.54)							
	Non-Hispanic White	299 (75.31)		156 (78.39)		143 (72.22)							
	Other	20 (5.04)		10 (5.03)		10 (5.05)							
**Geography, n (%)**											57.6 (1)		<.001
	Rural	2407 (12.04)		2020 (12.97)		387 (8.75)		N/A					

^a^N/A: not applicable.

### Comorbidities and Perceptions of Health

Overall, 40.18% (8036/20,000) of the patients reported being diagnosed with at least one comorbidity (Tables 3 and 4). Hypertension was the most commonly reported comorbidity (3535/20,000, 17.68%), followed by high cholesterol or triglycerides (3378/20,000, 16.89%), and diabetes or prediabetes (2276/20,000, 11.38%). Over 90% (18,363/20,000, 91.82%) of all patients reported that excess weight negatively affected their health, regardless of gender.

The disease burden was greater overall for patients with obesity compared with patients with preobesity. Approximately one-fifth (2793/13,754, 20.58%) of patients with obesity reported having hypertension or high cholesterol or triglyceride levels (2453/13,574, 18.07%). Almost half (8036/20,000, 40.18%) of the patients reported having one or more comorbidities, with significantly more patients with obesity compared with patients with preobesity reporting 1 or more comorbidities (*χ*^2^_1_=372.6; *P*<.001).

Females were less likely to report having at least one comorbidity (5992/15,576, 38.47% female vs 2044/4424, 46.20% male; *χ*^2^_1_=85.7; *P*<.001), and were significantly less likely to present with comorbidities other than osteoarthritis and gallbladder disease. Most of the comorbidities more likely to be reported by males carry increased cardiometabolic risk, including hypertension, high cholesterol, obstructive sleep apnea, and cardiovascular disease. Females and males similarly reported the belief that their weight negatively impacted their health (14,247/15,576, 91.47% female vs 4116/4424, 93.04% male; *χ*^2^_1_=11.3; *P*<.001).

**Table 3:** Sample health history by BMI category.

Health history variable		Total patients (N=20,000), n (%)	Patients with obesity (n=13,574), n (%)	Patients with preobesity (n=6426), n (%)	Chi-square (*df*)	*P* value
**Comorbidity type**						
	Hypertension	3535 (17.68)	2793 (20.58)	742 (11.55)	244.4 (1)	<.001
	High cholesterol	3378 (16.89)	2453 (18.07)	925 (14.39)	42.0 (1)	<.001
	Diabetes	2276 (11.38)	1796 (13.23)	480 (7.47)	143.6 (1)	<.001
	Gallbladder disease	1007 (5.04)	775 (5.71)	232 (3.61)	40.2 (1)	<.001
	Obstructive sleep apnea	1248 (6.24)	1102 (8.12)	146 (2.27)	254.8 (1)	<.001
	Osteoarthritis	848 (4.24)	610 (4.49)	238 (3.70)	6.7 (1)	.01
	Fatty Liver disease	517 (2.58)	439 (3.23)	78 (1.21)	70.7 (1)	<.001
	Cardiovascular disease	192 (0.96)	144 (1.06)	48 (0.75)	4.5 (1)	.03
≥1 comorbidity		8036 (40.18)	6079 (44.78)	1957 (30.45)	372.6 (1)	<.001
Believe weight negatively affects health		18,363 (91.82)	12,732 (93.80)	5631 (87.63)	220.8 (1)	<.001

**Table 4:** Sample health history by biological sex.

Health history variable		Total patients (N=20,000), n (%)	Female (n=15,576), n (%)	Male (n=4424), n (%)	Chi-square (*df*)	*P* value
**Comorbidity type**						
	Hypertension	3535 (17.68)	2433 (15.62)	1102 (24.91)	204.3 (1)	<.001
	High cholesterol	3378 (16.89)	2430 (15.60)	948 (21.43)	83.4 (1)	<.001
	Diabetes	2276 (11.38)	1736 (11.15)	540 (12.21)	3.8 (1)	.05
	Gallbladder disease	1007 (5.04)	922 (5.92)	85 (1.92)	115.2 (1)	<.001
	Obstructive sleep apnea	1248 (6.24)	649 (4.17)	599 (13.54)	517.4 (1)	<.001
	Osteoarthritis	848 (4.24)	759 (4.87)	89 (2.01)	69.5 (1)	<.001
	Fatty liver disease	517 (2.58)	356 (2.29)	161 (3.64)	25.1 (1)	<.001
	Cardiovascular disease	192 (0.96)	127 (0.82)	65 (1.47)	15.5 (1)	<.001
≥1 comorbidity		8036 (40.18)	5992 (38.47)	2044 (46.20)	85.7 (1)	<.001
Believe weight negatively affects health		18,363 (91.82)	14,247 (91.47)	4116 (93.04)	11.3 (1)	<.001

### Behavioral Characteristics

Almost all (19,732/20,000, 98.66%) patients had attempted at least one previous weight loss method, and half (10,067/20,000, 50.34%) of the patients had attempted 4 different weight loss methods (Tables 5 and 6). Overall, 28.76% (5752/20,000) of the patients reported previous use of prescription weight loss medications. Notably, 1.24% (247/20,000) of the patients in the full cohort reported prior bariatric surgery. This group had a mean BMI of 33.2 (SD 4.0) kg/m^2^ at baseline.

**Figure 2:**
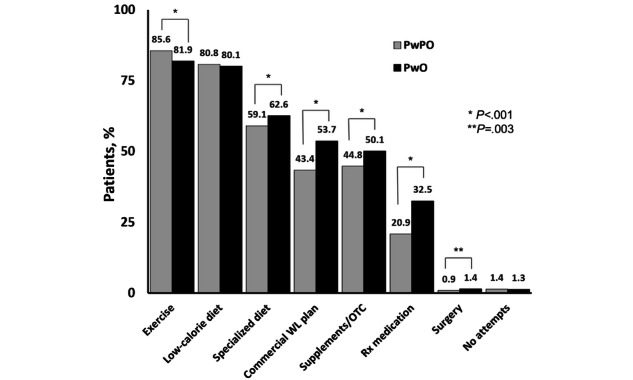
Types of weight loss (WL) attempts by BMI category. OTC: over-the-counter; PwPO: people with preobesity; PwO: people with obesity.

When comparing past weight loss behaviors across BMI groups, results showed that more patients with obesity reported having tried >4 weight loss methods compared with patients with preobesity (*χ*^2^_1_=110.1; *P*<.001; Figure 1). Exercise and a low-calorie diet were the most commonly attempted weight loss methods by both patients with preobesity and patients with obesity (Figure 2). More patients with obesity compared with patients with preobesity reported previous use of commercial weight loss plans (*χ*^2^_1_=185.2), specialized diets (omission of a category of food, *χ*^2^_1_=2 1.9), over-the-counter supplements (*χ*^2^_1_=50.8), and prescription weight loss medications (*χ*^2^_1_=283.3; all *P*<.001). Approximately one-third (4761/13,574, 35.07%) of patients with obesity reported engaging in aerobic exercise 3 or more days per week; 19.89% (2700/13,574) reported strength training of 3 or more days per week. Patients with preobesity were more likely to engage in aerobic (*χ*^2^_1_=183.2; *P*<.001) and strength training 3 or more times per week (*χ*^2^_1_=161.3; *P*<.001).

Females were more likely to have engaged in any and all past weight loss methods and were more likely to follow a specialized diet at the start of treatment. However, men were more likely to be engaged in strength training for 3 days per week (3256/15,576, 20.90% females vs 1238/4424, 27.98% males; *χ*^2^_1_=99.1, *P*<.001).

**Table 5:** Sample weight loss and dieting behavioral characteristics by BMI category.

Characteristic			Total patients (N=20,000), n (%)		Patients with obesity (n=13,574), n (%)		Patients with preobesity (n=6426), n (%)		Chi-square (*df*)		*P* value
**Number of methods tried**											
	≥1 WL^a^ method	19,732 (98.66)		13,393 (98.67)		6339 (98.65)		0.0 (1)		.96	
	≥2 WL methods	18,644 (93.22)		12,691 (93.49)		5953 (92.64)		5.1 (1)		.03	
	≥3 WL methods	15,172 (75.86)		10,498 (77.34)		4674 (72.74)		50.5 (1)		<.001	
	≥4 WL methods	10,067 (50.33)		7179 (52.88)		2888 (44.94)		110.1 (1)		<.001	
	≥5 WL methods	5434 (27.17)		4023 (29.64)		1411 (21.96)		130.0 (1)		<.001	
**Types of methods tried**											
	Exercise	16,613 (83.06)		11,113 (81.87)		5500 (85.59)		42.9 (1)		<.001	
	Low-calorie diet	16,072 (80.36)		10,879 (80.15)		5193 (80.81)		1.2 (1)		.28	
	Specialized diet	12,292 (61.46)		8493 (62.57)		3799 (59.12)		21.9 (1)		<.001	
	Commercial plan	10,085 (50.42)		7294 (53.74)		2791 (43.43)		185.2 (1)		<.001	
	Supplements or OTC^b^	9683 (48.42)		6807 (50.15)		2876 (44.76)		50.8 (1)		<.001	
	Rx WL medication	5752 (28.76)		4407 (32.47)		1345 (20.93)		283.3 (1)		<.001	
	Bariatric surgery	247 (1.24)		189 (1.39)		58 (0.90)		8.6 (1)		.003	
	No method	268 (1.34)		181 (1.33)		87 (1.35)		0.0 (1)		.96	
**Habits at treatment initiation**											
	Not following any diet	12,439 (62.20)		8623 (63.53)		3816 (59.38)		31.8 (1)		<.001	
	Aerobic exercise >3 days/week	7655 (38.27)		4761 (35.07)		2894 (45.04)		183.2 (1)		<.001	
	Strength training >3 days/week	4494 (22.47)		2700 (19.89)		1794 (27.92)		161.3 (1)		<.001	

^a^WL: weight loss.

^b^OTC: over-the-counter.

**Table 6:** Sample weight loss and dieting behavioral characteristics by biological sex.

Characteristic		Total patients (N=20,000), n (%)	Female (n=15,576), n (%)	Male (n=4424), n (%)	Chi-square (*df*)	*P* value
**Number of methods tried**						
	≥1 WL^a^ method	19,732 (98.66)	15,439 (99.12)	4293 (97.04)	112.9 (1)	<.001
	≥2 WL method	18,644 (93.22)	14,755 (94.73)	3889 (87.91)	253.7 (1)	<.001
	≥3 WL method	15,172 (75.86)	12,436 (79.84)	2736 (61.84)	609.3 (1)	<.001
	≥4 WL method	10,067 (50.33)	8663 (55.62)	1404 (31.74)	786.0 (1)	<.001
	≥5 WL method	5434 (27.17)	4884 (31.36)	550 (12.43)	623.5 (1)	<.001
**Types of methods tried**						
	Exercise	16,613 (83.06)	12,880 (82.69)	3733 (84.38)	7.0 (1)	.008
	Low-calorie diet	16,072 (80.36)	12,797 (82.16)	3275 (74.03)	144.3 (1)	<.001
	Specialized diet	12,292 (61.46)	9757 (62.64)	2535 (57.30)	41.5 (1)	<.001
	Commercial plan	10,085 (50.42)	8943 (57.42)	1142 (25.81)	1376.4 (1)	<.001
	Supplements or OTC^b^	9683 (48.42)	8030 (51.55)	1653 (37.36)	277.8 (1)	<.001
	Rx WL medication	5752 (28.76)	5216 (33.49)	536 (12.12)	768.1 (1)	<.001
	Bariatric surgery	247 (1.24)	209 (1.34)	38 (0.86)	6.6 (1)	.01
	No method	268 (1.34)	137 (0.88)	131 (2.96)	112.9 (1)	<.001
**Habits at treatment initiation**						
	Not following any diet	12,439 (62.20)	9442 (60.62)	2997 (67.74)	74.4 (1)	<.001
	Aerobic exercise >3 days/week	7655 (38.27)	5939 (38.13)	1716 (38.79)	0.6 (1)	.44
	Strength training >3 days/week	4494 (22.47)	3256 (20.90)	1238 (27.98)	99.1 (1)	<.001

^a^WL: weight loss.

^b^OTC: over-the-counter.

### Access to Care

A total of 21.26% (4252/20,000) of the patients lived in a county that was designated as a primary care HPSA in its entirety. Approximately 29.25% (5850/20,000) of patients lived in the same zip code as an ABOM specialist, and most lived in the same county as an ABOM specialist (Tables 7 and 8).

Comparing across categories, patients with preobesity and females were slightly more likely to live in a whole-county primary care HPSA and slightly less likely to live in the same zip code or county as an ABOM specialist compared with patients with obesity.

**Table 7:** Sample access to care by BMI category.

Characteristic			Total patients (N=20,000), n (%)		Patients with obesity (n=13,574), n (%)		Patients with preobesity (n=6426), n (%)		Chi-square (*df*)		*P* value
**HPSA^a^ category, county of residence, n (%)**									40.9 (1)		<.001
	None of county is shortage area	1634 (8.20)		1102 (8.15)		532 (8.32)					
	Part of county is shortage area	14,034 (70.45)		9364 (69.23)		4670 (73.03)					
	Whole county is shortage area	4252 (21.35)		3059 (22.62)		1193 (18.66)					
Has obesity specialist in zip code			5850 (29.25)		3825 (28.18)		2025 (31.52)		23.5 (1)		<.001
Has obesity specialist in county			17,030 (85.15)		11,437 (84.26)		5593 (87.05)		26.9 (1)		<.001

^a^HPSA: health care provider shortage area.

**Table 8:** Sample access to care by biological sex.

Characteristic		Total patients (N=20,000), n (%)	Female (n=15,576), n (%)	Male (n=4424), n (%)	Chi-square (*df*)		*P* value	
**HPSA^a^ category, county of residence**						25.7 (1)		<.001
	None of county is shortage area	1634 (8.20)	1289 (8.30)	345 (7.86)				
	Part of county is shortage area	14,034 (70.45)	10,810 (69.62)	3224 (73.41)				
	Whole county is shortage area	4252 (21.35)	3429 (22.08)	823 (18.74)				
Has obesity specialist in zip code		5850 (29.25)	4374 (28.08)	1476 (33.36)	46.4 (1)		<.001	
Has obesity specialist in county		17,030 (85.15)	13,060 (83.85)	3970 (89.74)	94.4 (1)		<.001	

^a^HPSA: health care area shortage area.

## Discussion

### Principal Findings

To the best of our knowledge, this report constitutes the largest published cohort of DTC telehealth users seeking prescriptive weight management. The size of this sample of patients (20,000) who sought and obtained OSH on a web-based DTC platform supports the notion that telehealth appears to be a viable means of delivering prescription weight management interventions nationwide. Approximately one-third of the patients in our sample had preobesity (ie, BMI 25-30 kg/m^2^) when they first initiated treatment with OSH, and most patients in this cohort reported no weight-related comorbidities. In addition to OSH, recommended treatment options for patients in this BMI category are limited to lifestyle modification and orlistat, an over-the-counter weight loss treatment. Our data suggest that people with preobesity do seek prescription weight management care as it becomes available to them. Given the chronic, progressive nature of obesity and the willingness of patients with preobesity to seek care, additional tools such as OSH and improved access through DTC telehealth are important opportunities for early intervention.

Almost all patients (19,732/20,000, 98.66%) in our large cohort reported previously attempting at least one weight loss method before OSH. Surprisingly, almost one-third of the patients (5752/20,000, 28.76%) had previously used prescription weight loss medication. This is much higher than the ~2% of eligible patients who are normally prescribed weight loss medication, suggesting that people in our cohort had a greater predisposition to this type of treatment and were therefore more aware or willing to try additional prescriptive therapy [7-9]. About half of our cohort (including 2876/6426, 44.76% of patients with preobesity) also reported previous use of weight loss supplements and over-the-counter treatments. Dietary weight loss supplements are generally not recommended given the lack of evidence supporting their safety and efficacy. The fact that half of our cohort (9683/20,000, 48.42%) had previously used these unproven therapies further emphasizes the need for accessible and evidence-based treatment options.

In addition, we examined age, sex, and geographic trends in our cohort. In our sample, approximately 10% of patients were aged ≥60 years, with the oldest being 86 years, supporting the idea that DTC telehealth is a viable treatment option among older patients. This is consistent with recent studies that demonstrated that older patients commonly utilized telehealth for ambulatory visits during the COVID-19 pandemic [19]. These findings are contrary to the concerns that older adults lack the awareness, experience, and confidence to successfully navigate technology [20,21].

In our cohort, women were more likely to initiate OSH and had a lower BMI than men. Previous studies have similarly demonstrated that men are less likely to engage in weight loss therapies than are women [22]. Interestingly, both males and females in our cohort similarly believed that weight negatively affected their health (>90% of each group). This observation contrasts with that of a large survey, which suggested that only about half of people with obesity are concerned with the impact of excess weight on health [23]. This may be because of the differences in the 2 groups: our cohort-initiated treatment, which demonstrates a level of self-awareness and readiness to take action, versus the survey respondents comprised of the general population.

Finally, we found that most (17,030/20,000, 85.15%) patients were located in the same county as an ABOM-certified diplomate provider. This is consistent with a recent study that showed that the median travel time for a patient to reach an obesity medicine provider was approximately 10 minutes. Despite this, access to an obesity specialist is still hindered, in part because of the shortage of obesity providers relative to people with obesity (1 provider for every 20,000 patients). There may also be other reasons that a patient who lives near an obesity medicine provider elected to use the telehealth weight management platform instead, including the convenience of DTC telehealth, previous experience of weight stigma and bias, extensive waitlists for new patient appointments, lack of patient awareness of nearby obesity specialist providers, and less complex care requirements.

### Practice Implications

Given the prevalence of preobesity and obesity, technology that can provide evidence-based weight management at scale is a valuable tool. Obesity is a disease that is particularly well-positioned for management via DTC telehealth. First, two-thirds of people with obesity reported experiencing weight stigma from their providers. This results in greater health care avoidance, lower frequency of routine checkups, and lower quality of health care [13]. Most people with obesity (>80%) actually believe that weight management is their sole responsibility, whereas three-quarters of health care providers think it is *their* responsibility as the provider to manage weight [10]. Ironically, health care providers rarely discuss weight loss during routine appointments, with the most common reasons being insufficient time, more important issues to discuss, and lack of belief that the patient is motivated to lose weight; the latter reason is in and of itself an example of bias [10]. Finally, treatment options exist for weight management with easily understood mechanisms of action and patient-centric routes of administration that are well suited for prescribing via telemedicine [6].

Early intervention at scale on web-based platforms may also play an important role in shifting the weight management paradigm toward the *prevention* of obesity, rather than just the *treatment* of obesity. People with preobesity represent an important group for targeted treatment to prevent the progression to obesity and its related comorbidities [24]. On an average, people gain approximately 1 pound per year as they age [25]. Without interventional treatment, adults with preobesity continue to gain weight, moving steadily into obesity and sometimes severe obesity categories [26]. Therefore, early intervention in weight management (ie, in patients with preobesity) is recommended to prevent disease progression to obesity [24]. However, weight loss interventions, including pharmacotherapy, are inadequately used today for obesity prevention and most are not approved for use until a patient has a higher preobesity BMI range (≥27 kg/m^2^) with at least one weight-related comorbidity. A recent large-scale review of electronic health record data determined that less than 0.4% of eligible people with preobesity received pharmacotherapy for weight loss [8]. However, most Food and Drug Administration–approved weight loss medications are indicated for use in people with preobesity who have at least one weight-related comorbidity (eg, diabetes). As demonstrated by the high proportion of people with preobesity in our dataset, DTC telehealth platforms and early interventional treatments such as OSH may reduce some of the existing barriers to care for these patients, helping shift the focus of management from treatment to prevention.

The acceptability and availability of telehealth has changed in response to the pandemic. Demonstrated telehealth benefits include connectivity, cost efficiency, improved access, medication adherence, and decreased hospitalizations [27,28]. In light of the findings of our study, DTC telehealth can help address issues such as provider shortages and the need for expanded geographical areas. Treating preobesity and obesity via DTC telehealth could support patients with improved cost efficiencies such as reduced travel to appointments, decreased work absenteeism, and improved workday efficiency. All of these can translate to lower health care costs for patients, employers, and insurers, and most importantly, improved provision of care.

### Limitations

Although our analysis provides valuable insights into the characteristics of people using telehealth for weight management, there are several limitations that should be noted. First, all data were self-reported by the patients, and errors in patient recall or reporting could have skewed our results. For example, self-reported weight tends to underestimate actual weight; in this cohort, this may have skewed our population toward a slightly higher proportion of patients with preobesity [29]. Similarly, the rate of hypertension reported in our patient population was lower than that reported in the general population, possibly because of underreporting or errors in patient recall [30]. Incorporating objective methods of data collection via remote patient monitoring opportunities (eg, a wireless weight scale) would help reduce the self-reporting bias in the future. Second, our cohort consisted only of patients who were eligible to receive a prescription for OSH; therefore, people with BMI <25 kg/m^2^ or >40 kg/m^2^ were excluded, which could affect the generalizability of our observations. This may be important in populations where preobesity and obesity are diagnosed at a lower BMI, secondary to an associated increased cardiometabolic risk. Our telehealth patient population was gender-imbalanced, and while weight loss interventions are typically weighted toward female patients, this could also affect generalizability [31]. Questions about race and ethnicity were not initially included in the baseline telehealth questionnaire; therefore, not all the patients in our cohort reported this information. Future analyses could be stratified by baseline demographics to ensure adequate and balanced representation. In addition, while patient proximity to ABOM-certified providers was used as a surrogate measure of access to obesity medicine specialist care, it is important to note that non-specialist providers can prescribe obesity treatment. Finally, some of the practice site locations for ABOM-certified providers were obtained using a manual web search, introducing the possibility that an incorrect or secondary practice site was used incorrectly for some providers.

### Conclusions

Our analysis supports the use of telehealth as a means of providing prescriptive weight management treatment to populations seeking care. Patients with preobesity are undertreated and actively seeking new weight management options. Future cross-sectional studies are needed to determine whether these patient characteristic trends continue once DTC telehealth is more broadly used for weight management.

## Abbreviations

ABOM: American Board of Obesity Medicine
DTC: direct-to-consumer
HPSA: healthcare provider shortage area
OSH: oral superabsorbent hydrogel

## Data Availability

Owing to the nature of this research and its use of proprietary information, supporting data are not readily available for sharing. However, requests from qualified researchers who engage in rigorous, independent scientific research and are willing to enter into a formal data use agreement may be considered on a case-by-case basis. For more information, please contact research@ro.co.
